# STRESSOR-RELATED PERSEVERATIVE COGNITION PREDICTS MORTALITY RISK IN MIDLIFE AND OLDER AGE

**DOI:** 10.1093/geroni/igae098.1975

**Published:** 2024-12-31

**Authors:** Robert Stawski, Jacqueline Mogle, Dakota Witzel

**Affiliations:** University of Essex, Colchester, England, United Kingdom; Clemson University, Clemson, South Carolina, United States; South Dakota State University, Brookings, South Dakota, United States

## Abstract

Perseverative cognition (e.g., worry and rumination) has been highlighted as a key indicator of stress and a mechanism linking stress to health and mortality risk. Despite this, research linking daily stress to mortality has focused on affective pathways (e.g., affective reactivity), largely ignoring the role of perseverative cognition in daily stress-mortality associations during midlife and older age. We address this gap by leveraging data from the National Study of Daily Experiences Refresher cohort (2012-2014). Respondents (N=694, Mage=47.5, SD=12.6, Range=25-75; 57% women) completed telephone interviews assessing perseverative cognition, negative affect, and the daily stressors on eight consecutive evenings. Decedents (n=15) date and cause of mortality, through 2019, were obtained from the National Death Index. Multilevel models were used to derive cognitive reactivity slopes – reflecting stressor-related increases in perseverative cognition - which were subsequently used in Cox proportional hazards regression models to predict survival. Results indicated higher levels of average daily perseverative cognition (on non-stressor days) were associated with a two-fold increase in mortality risk (Hazard Ratio=2.04, p=.007), while larger stressor-related increases in perseverative cognition (i.e., cognitive reactivity) were associated with an 81% increase in mortality risk (Hazard Ratio=1.81, p=.002). Findings were robust to adjustment for age, sex, differential stressor exposure, daily negative affect, and affective reactivity to daily stressors. Discussion will focus on why daily perseverative cognition is important for understanding health and wellbeing and represents an important intervention target to reduce health and mortality risk during midlife and older age.

